# *Blochmannia *endosymbionts improve colony growth and immune defence in the ant *Camponotus fellah*

**DOI:** 10.1186/1471-2180-9-29

**Published:** 2009-02-06

**Authors:** Danival J de Souza, Annie Bézier, Delphine Depoix, Jean-Michel Drezen, Alain Lenoir

**Affiliations:** 1 Institut de Recherche sur la Biologie de l'Insecte, UMR CNRS 6035, Université François Rabelais, Avenue Monge, Parc de Grandmont 37200, Tours, France; 2 Departamento de Biologia Animal, Universidade Federal de Viçosa, Viçosa MG 36570-000, Brazil; 3 Laboratoire de Biologie Fonctionnelle des Protozoaires, USM504-EA3335, Département Régulations, Développement, Diversité Moléculaire, Muséum National d'Histoire Naturelle, 61 rue Buffon, 75005 Paris, France

## Abstract

**Background:**

Microorganisms are a large and diverse form of life. Many of them live in association with large multicellular organisms, developing symbiotic relations with the host and some have even evolved to form obligate endosymbiosis [1]. All Carpenter ants (genus *Camponotus*) studied hitherto harbour primary endosymbiotic bacteria of the *Blochmannia *genus. The role of these bacteria in ant nutrition has been demonstrated [2] but the omnivorous diet of these ants lead us to hypothesize that the bacteria might provide additional advantages to their host. In this study, we establish links between *Blochmannia*, growth of starting new colonies and the host immune response.

**Results:**

We manipulated the number of bacterial endosymbionts in incipient laboratory-reared colonies of *Camponotus fellah *by administrating doses of an antibiotic (Rifampin) mixed in honey-solution. Efficiency of the treatment was estimated by quantitative polymerase chain reaction and Fluorescent *in situ *hybridization (FISH), using *Blochmannia *specific primers (qPCR) and two fluorescent probes (one for all Eubacterial and other specific for *Blochmannia*). Very few or no bacteria could be detected in treated ants. Incipient Rifampin treated colonies had significantly lower numbers of brood and adult workers than control colonies. The immune response of ants from control and treated colonies was estimated by inserting nylon filaments in the gaster and removing it after 24 h. In the control colonies, the encapsulation response was positively correlated to the bacterial amount, while no correlation was observed in treated colonies. Indeed, antibiotic treatment increased the encapsulation response of the workers, probably due to stress conditions.

**Conclusion:**

The increased growth rate observed in non-treated colonies confirms the importance of *Blochmannia *in this phase of colony development. This would provide an important selective advantage during colony founding, where the colonies are faced with severe inter and intraspecific competition. Furthermore, the bacteria improve the workers encapsulation response. Thus, these ants are likely to be less susceptible to various pathogen attacks, such as the Phoridae fly parasitoids, normally found in the vicinity of *Camponotus *nests. These advantages might explain the remarkable ecological success of this ant genus, comprising more than 1000 species.

## Background

An increasing set of data is shedding light on the role of microorganisms that have co-evolved with their hosts, including humans [3]. They illustrate the high diversity of endosymbiotic forms among living organisms. Moreover the evidence of gene transfer between bacterial cells or viruses and eukaryotic cells supports the theory of symbiotic relationships as a major force driving evolution [4] and as a source of phenotypic complexity [5]. Multiple new symbionts are regularly discovered in the same host, which can compete or cooperate [3,6]. Normally, they play a role in host nutrition; defence against pathogens remains an underappreciated benefit of such associations, both in invertebrates and vertebrates [7,8]. Social insects are particularly concerned as they are highly susceptible to infectious diseases, due to their lifestyle, and have evolved several associations with microorganisms [9].

Endosymbionts are very common among insects, especially in those sucking plant sap, feeding on vertebrate blood for their entire life span, and those that eat wood and keratin. As they are all strict specialists in nourishment, it is assumed that endosymbionts play a role in providing complementary elements absent from these restricted diets. *Camponotu*s genus, carpenter ants, have established an association with intracellular endosymbionts *Blochmannia*, a taxon of γ-Proteobacteria, found in all *Camponotus *species studied hitherto [10]. The bacteria live within specialized cells, the bacteriocytes. The function of the endosymbionts is not fully elucidated but their role as dietary complement suppliers has been pointed out after the genome sequence analysis of two *Blochmannia *species. The bacteria is probably able to supply nitrogen and sulphur compounds to the host [11-13]. Moreover, bacteria elimination using antibiotic treatment is deleterious and chemically defined diets can complement bacteria suppression [2,14] demonstrating the necessary nutritional role of bacteria. However, the presence of *Blochmannia *in omnivorous *Camponotus *species suggests that bacteria may also have other functions beneficial to the ants. Some studies have suggested that *Blochmannia *may play a more important role during the colony founding phase and growth rather than in adult worker maintenance [15] or may play a role in pheromone production [16].

Microbes that forms chronic infections in a host lineage may evolve to promote host survival or benefits to its host, as this will help to maintain its immediate ecological resource [17]. In this context, secondary endosymbionts can provide hosts with defences against parasites, beyond nutritional advantages [18,19]. So far, no similar example with primary endosymbionts has been reported. Externally located bacteria are also capable of conferring protection to insect hosts against parasite infections [20].

Here, we tested the hypotheses that *Blochmannia *provide faster colony development in the initial stages (incipient colonies) as previously stated [15] and/or improve the host immune system of the host. We used the encapsulation rate as an index of the immune response and analysed whether it was correlated or not with the number of bacteria. The use of incipient colonies, obtained from founding queens, is a suitable choice since it allows the study of animals of similar ages and reduces the effects of natural selection operating on colonies throughout their development.

## Results

### Endosymbiont identification

The 16S rDNA endosymbiont sequence was deposited in the GenBank database under accession number EF422835. According to the Ribosomal Database Project [21], the 16S rDNA sequence of *Camponotus fellah *endosymbiont correspond to an unclassified γ-Proteobacteria closely related to 16rDNA sequences from *Blochmannia *endosymbionts bacteria of various *Camponotus *ant species. This sequence has G+C content of 47% which is near to that of other *Blochmannia *symbionts.

When compared with the nucleic sequences of other *Blochmannia *(tools available in NCBI/Blast), maximum identity ranged from 91–93%. However, other *Blochmannia *species present in GenBank exhibit up to 98% of identity to each other. Phylogenetic comparisons showed the existence of a monophyletic group containing classified and unclassified endosymbionts from *Camponotus *ant species, closer to other insect endosymbionts and distinct from other outgroup bacteria (data not published).

The use of FISH with primers specific for Eubacteria and *Blochmannia *endosymbionts showed that bacteriocytes of midgut preparations were full of bacteria. In these preparations it was possible to see the individual bacterium and its rod form. The bacteriocytes were also detected in the oocytes by FISH as well.

### Effectiveness of antibiotic treatment

**Figure 1:**
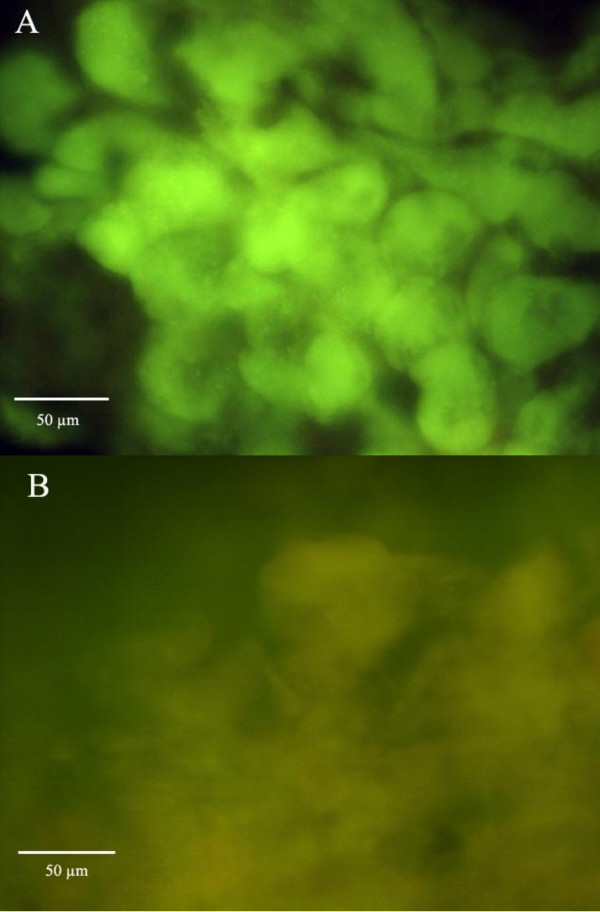
***Blochmannia *specific fluorescent *in situ *hybridisation (FISH) of bacteriocytes (green) in *C. fellah *control worker (A) and Rifampin treated worker midguts (B)**. The bacteriocytes of treated worker are hardly visible.

**Figure 2:**
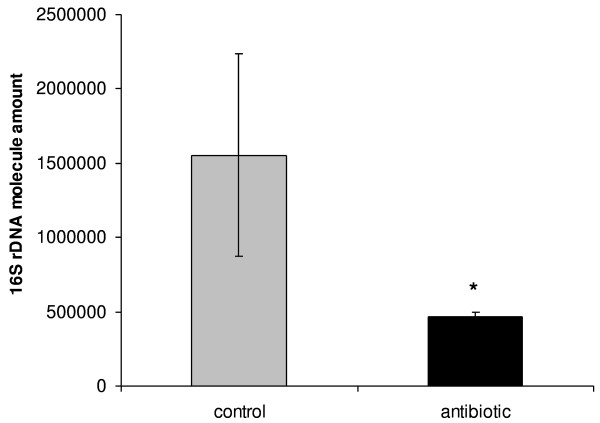
**Endosymbiont number estimation in worker midguts, after 3 months of antibiotic treatment**. Workers from treated groups present a mean number of bacteria significantly lower than the control group (Mann-Whitney's U-test = 179.00, Z = -3.48, p < 0.001). The bars represent the mean number of 16S rDNA molecules ± semi-quartile range.

The quantity of *Blochmannia *in midgut bacteriocytes was estimated after Rifampin treatment using two complementary methods: real-time quantitative PCR and Fluorescent *in situ *hybridization (FISH). The two methods showed a reduction of *Blochmannia *numbers in midgut bacteriocytes after 12-weeks of antibiotic treatment. Within this period, FISH did not detect the presence of *Blochmania *in the bacteriocytes (Fig. 1). However quantitative real-time PCR indicated that the bacteria were not completely eliminated as a low quantity of 16S rDNA bacteria molecules can be detected in the midgut. Treated and control groups differed significantly in their content of *Blochmannia *measured as 16S rDNA molecules (Mann-Whitney's U-test = 179.00, Z = -3.48, p < 0.001) (Fig. 2). The treatment reduced the quantity of bacteria by 75%. Moreover, the individual variation in bacteria amount was more constant in antibiotic treated colonies than in control colonies.

### Evaluation of colony development

**Figure 3:**
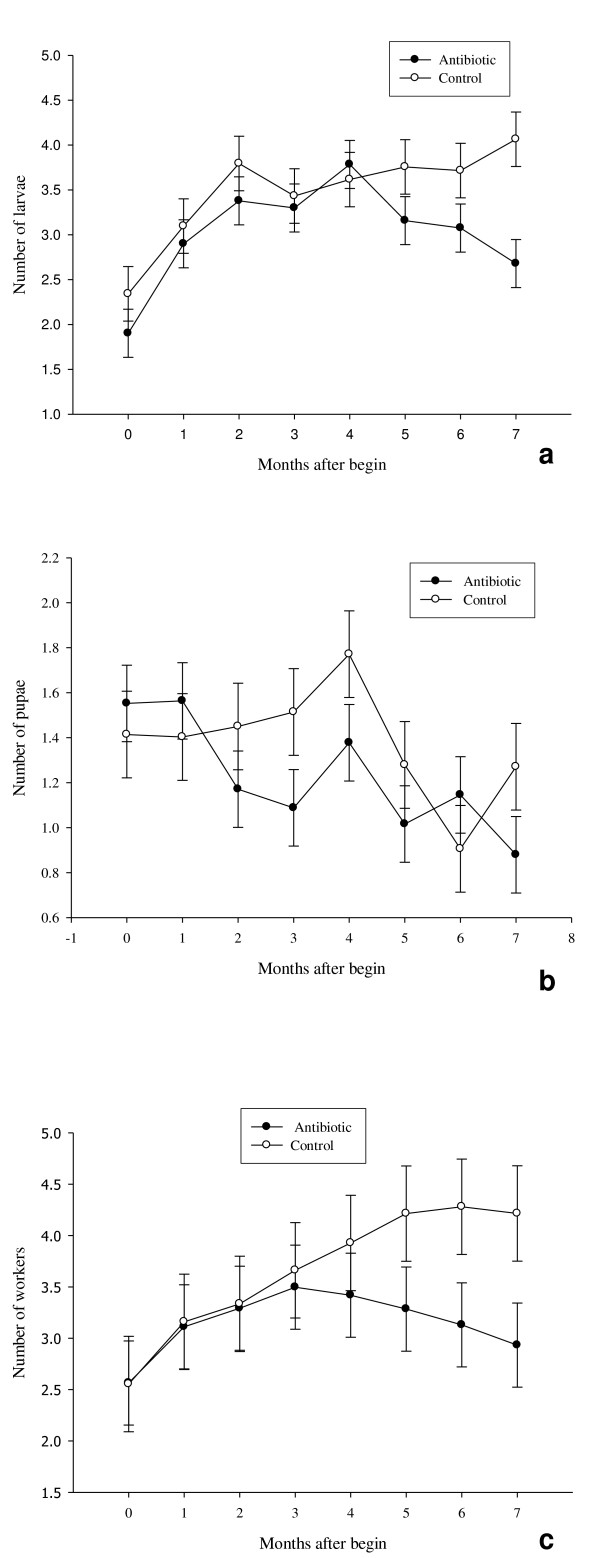
**Mean number of larvae (a), pupae (b) and workers (c), square-root transformed (± SE), for control and antibiotic-treated colonies. N = 7 and 9, respectively**.

Each colony was composed of at least one larva, pupa or worker and queen. Colonies composed only with the queen or colonies with a dying queen during the experiment were excluded. After seven months, seven control colonies and nine treated colonies were kept for further analysis. Workers, larvae and pupae numbers were not significantly different during the first three months after the beginning of the experiments. After this time, untreated colonies displayed more accentuated larvae production and had a higher number of adult workers (Fig 3a and 3c, see table 1, for all statistical results). Pupae number varied significantly throughout the time of the experiment but no difference between treated and control colonies was observed (Fig 3b). The variation in workers numbers was significatively different between treated and control colonies with untreated colonies having more workers (Fig 3c).

**Table 1:** 

ANOVA main effects			
Mean number	Antibiotic × control	Time	Interaction
larvae	F_1,112 _= 10.12**	F_7,112 _= 6.08***	F_7,112 _= 0.26
pupae	F_1,112 _= 2.79	F_7,112 _= 2.52*	F_7,112 _= 1.20
workers	F_1,112 _= 5.53*	F_7,112 _= 1.69	F_7,112 _= 0.75

Mean number of larvae, pupae and workers analysed by ANOVA. Significance levels are *P < 0.05, **P < 0.01 and ***P ≤ 0.001.

### Amount of *Blochmannia *endosymbiont versus encapsulation response

**Figure 4:**
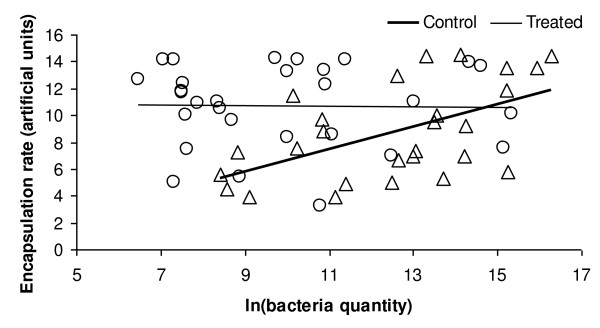
**Relationship between *Blochmannia *endosymbiont amounts, expressed as ln of 16S rDNA molecules for individual midgut, and encapsulation response**. Δ represent workers from untreated colonies and O represent treated workers.

When expressing encapsulation rate versus 16S rDNA molecules amount (as measure of *Blochmannia *amount in individual midgut), control and treated colonies displayed different patterns of immune response. We found a significant positive correlation between encapsulation rate and bacteria amount in the ants from control colonies: the bacteria did facilitate the encapsulation response (Pearson's r, p = 0.003, n = 27, Fig. 4). On the contrary, ants from treated colonies did not display a correlation between the amount of bacteria in the midgut and the encapsulation response (Pearson's r, p = 0.92, n = 29, Fig. 4). Thus, it seems that antibiotic treatment eliminated the bacterial effects on the immune encapsulation response. An ANCOVA analysis with the encapsulation rate as independent variable showed that treated workers present a significant increase in encapsulation rate (F_1,53 _= 8.61, p = 0.005). The regression inclinations of treated and control groups differed significantly (F_1,52 _= 10.06, p = 0.003).

## Discussion and Conclusion

In this study, we confirmed that *Blochmannia *plays an important role for *Camponotus *ants by improving the colony growth. We also demonstrated for the first time that *Blochmannia *interacts with the ant immune defence.

Antibiotic treatment with Rifampin considerably reduced the endosymbiont number in the midgut, although they were never totally eliminated and there was a great variability between workers. This may be due to different access to the antibiotic and some ants may not drink the antibiotic solution or, as observed by Feldhaar et al. (2007), may be explained by the fact that DNA of the endosymbiont may still be detectable by qRT-PCR when bacteria are not alive or active. Additionally, it was confirmed that bacterial sequences were not integrated in the genome of the ant by a PCR test performed on ant DNA from legs using *Blochmannia *16S rDNA and ant 18S rDNA primers (data not shown).

The treatment had a remarkable impact on colony development by reducing larvae production and worker numbers, corroborating previous worker [2]. Carrying out the studies in entire incipient colonies, we can demonstrate the importance of endosymbionts in this phase of colony development. According Feldhaar et al. (2007), essential amino acids provided by endosymbionts improve workers ability to raise pupae. Here, we have verified that control colonies exhibited a bigger population in the first seven months of colony development. Since the establishment phase is critical for new colonies, harbouring more bacteria may have major ecological consequences in a context of inter and intraspecific competition: more workers confers a special advantage to maintain a young colony, occupy and monopolize food resources. Indeed, animal protein food resources are more unpredictable in the time-space scale. *Blochmannia *presence could signify a possible adaptation for ants to fluctuations in protein availability, permitting the colony growth even in absence of preys. We do not know the mechanisms allowing an increase in brood production, beyond the direct nutritional effects on treated queen, but several mechanisms are plausible, including a direct oogenesis control. For example, it has been demonstrated that *Wolbachia *bacteria are necessary for the host oogenesis in a particular strain of the parasitic wasp *Asobara tabida *[22]. Furthermore, it was evidenced that apoptosis prevention of nurse cells by *Wolbachia *can regulate the host oogenesis [23].

We have demonstrated that *Blochmannia *play another important function by improving *Camponotus *host immune system. The encapsulation rate measured in Rifampin treated workers was significantly higher when compared with control colonies. Although no evident toxic effect (like increase in mortality) was observed, it is expected that antibiotic treatment has stressing effects on workers and that the increase of the encapsulation rate might correspond to an adaptive response to stress. Furthermore, antibiotic treatment seemed to mask the effects of endosymbiont number on encapsulation response observed in control colonies, where the bacteria favoured the encapsulation response. Positive effects of symbionts on host immune system have been described in the last years. For example, the facultative symbionts of *Acyrthosiphon pisum *(the pea aphid) confer it resistance to parasitoid attacks [18]. Recently, it has been demonstrated that *Wolbachia *confer vigorous antiviral protection to *Drosophila *[19]. The mechanisms by which the resistance is expressed is still unknown, but in another example it was showed that symbiotic bacteria could compete directly for space and resources and thus prevent host colonization by pathogens [24,25].

Encapsulation is the principal physiological response against parasitoids suggesting an important role of the stimulation induced by *Blochmannia *in the protection against parasites. This strong interaction between symbiotic bacteria and ants may explain the persistence and broad occurrence of symbiotic bacteria in the *Camponotu*s genus. Ants from *Camponotus *genus are abundant almost everywhere in the world where ants are found, comprising more than 600 described species within an estimated number greater than 1,000 species [26]. Its large distribution, the diversity of forms and food behaviour and the occurrence on diverse environments make the system *Camponotus/Blochmannia *an interesting model to study how ecological forces determine symbiont characteristics and how bacteria determine the ant traits. For example, it is interesting to determine how genetic differences found among different species of *Blochmannia *could be related to host ecological characteristics.

The social habits of the ants make them particularly vulnerable to several parasites and parasitoids. Phoridae flies are frequently found around *Camponotus *nests and their influence is fundamental in regulating the ant communities [27]. So, it can be expected that *Camponotus *species more exposed to Phoridae attack should harbour more bacteria. The physiological mechanism linking bacterial amount and encapsulation response remains unknown. Although the better workers "quality" due to extra nutrients furnished by bacteria is the more probable explanation, direct production of biomolecules in stress situation should not be excluded.

An efficient immune system is a major trait allowing the existence of social insect colonies with thousand of individuals, genetically related [28], living close together, constantly exposed to parasitic disease risks. Competition in the first stages of colony growth constitutes also a great challenge to reach the reproductive stage. Thus, *Blochmannia *endosymbionts appear to be a fundamental partner, responsible for the ecological success of *Camponotus *ants. As more than 10% of insect species depend on obligate bacterial mutualists for their viability and reproduction [29], the research on symbiosis between bacteria and animals appears to be a new and promising field, particularly in social insects.

## Methods

### *Camponotus fellah*: sampling sites and culture

*Camponotus *ants develop by complete metamorphosis, like all hymenopterans, going through stages of the egg, larva, pupa, and adult worker or reproductive. Pupae exist in conspicuous silk cocoons. Newly fecundated females start a new colony, caring for their first brood of larvae until they develop into workers, which then begin to forage for food. Founding queens of *C. fellah *were collected in Tel-Aviv in March 2006 and 2007. Colonies were kept in plastic containers (20 × 20 × 10 cm) with plaster nests in a climate chamber (constant temperature of 28°C, 12 h light per day), and were fed twice a week with *Tenebrio molitor *larvae and commercial honey solution (BeeHappy^®^, France). In 2006 and 2007 we used 10 control colonies (fed with *Tenebrio *and honey) and 10 treated colonies (fed with *Tenebrio *and honey in the first week, and *Tenebrio *larvae and honey solution containing 1% of the antibiotic Rifampin the second week and after). In previous studies on other *Camponotus *species [30] Rifampin was shown to reduce the number of bacteria without increasing mortality and did not cause damage to the ant midgut tissues. The treatment was maintained during three months.

Because the occurrence of *Wolbachia *is widespread in ants [31] and these symbiotic bacteria can have negative effects on immunity-related traits of insects [32], their incidence was checked in the *C. fellah *colonies studied, using two pairs of primers based on *Wolbachia ftsZ *sequences [31], so as to amplify A and B-group *Wolbachia *specific product [31]. No incidence of *Wolbachia *was detected.

### Symbiont identification

Symbiont identification was based on sequencing of the *16S rRNA *gene and Fluorescent *in situ *hybridization. The *16S rRNA *gene was amplified using the previously described primers SL (TTGGGATCCAGAGTTTGATCATGGCTCAGAT) and SR (CACGAATTCTACCTTGTTACGACTTCACCCC) [33]. The PCR reactions were performed in a total volume of 25 μl containing 2.5 mM dNTPs, 7.5 mM MgCl2, 5 pmol each oligonucleotide and 2.5 U/μl *Taq *DNA polymerase (GoldStar^®^). Amplification was performed in an Eppendorf thermocycler according to the following conditions: 30 s denaturation at 94°C, 30 s primers annealing at 55 °C and 1.5 min primer extension at 72°C, running 35 cycles. The amplified DNA fragment of approximately 1,550 bp was purified using a QIAquick PCR purification Kit (Qiagen) and directly sequenced using the ABI PRISM™ dye terminator cycle. The sequencing reactions were performed using the SL and SR primers and using the two internal primers sequences CampL (5'-GAATTACTGGGCGTAAAGAGT-3') and CampR (5'-GGAACGTATTCACCG TGAC-3'). Additionally, two reverse primers were designed to complete the sequences: P1rev(5'-CTCTCAGACCAGCTAAGGAT-3') and P2rev(5'-ACCGCTACACCTGGAATTCT-3').

The oligonucleotides used for *in situ *hybridization were described previously. Bacteriocytes were visualized by FISH with oligonucleotide probes Eub338 (5'-GCTGCCTCCCGTAGGAGT-3') [34], targeting a conserved region of the eubacterial 16S rRNA, and with Bfl172 (5'-CCTATCTGGGTTCATCCAATGGCATAAGGC-3'), targeting a 16S rRNA region specific for *B. floridanus *[33]. Probes were labelled with the fluorescent dyes Cy3 or FITC at the 5' end (MWG-BIOTECH AG, Ebersberg, Germany). For protocol process details see [2]. The ovaries of three years old queen were dissected, fixed and hybridized like the midguts. The slides were analyzed with a Leica DMR microscope (Leica Microsystems, Wetzlar, Germany) and pictures were taken with a RT Slider digital camera (Diagnostic Instruments Inc., Sterling Heights, MI, USA).

### Evaluation of colony development

Colonies collected in 2006 were used to evaluate control colonies *versus *treated colonies development. Over a period of seven months (including the first three months of antibiotic treatment) the number of brood (larvae and pupae) and workers in each colony were counted each month, during seven months.

### Encapsulation rate assay

Encapsulation followed by melanisation is an efficient innate immune response against parasites. We can trigger this response by inserting an inert antigen, like nylon filament. To measure the ant immune response, an encapsulation test was performed by inserting a 1.5 mm-long piece of nylon monofilament (0.12 mm diameter) in the pleural membrane between the second and third tergite. This procedure was carried out on three workers from each colony, with a total of 30 workers for each group, based on the procedures adopted by Rantala & Kortet [35]. Twenty four hours after, the implants were removed from the haemocoel and placed on a glass slide to be mounted into Clarion™ medium. The filament was examined under a light microscope and photographed using a digital camera (Olympus DP50). The mean grey value of the whole implant was measured using the ImageJ 1.37v software. We assumed that the darkest grey received the highest encapsulation rate (total black). The background grey value was subtracted to correct the values of the implants. The midgut of each worker was dissected in sterile PBS (137 mM NaCl-2.7 mM KCl-4.3 mM sodium phosphate-1.4 mM potassium phosphate, pH 7.2) and conserved in tubes independently at -20C° for quantitative PCR.

### Assessing antibiotic treatment effects

Antibiotc treatments effects were assessed by two different and complementary techniques: Real time qPCR and Fluorescent *in situ *hybridization (Fish).

#### Real time qPCR

DNA was isolated from the whole midgut of individual workers that were previously tested for encapsulation rate (see Encapsulation assays) using DNA extraction commercial kit (Gentra Systems Puregene^©^, Minneapolis, MN, USA) according to manufacturer's recommendations then resuspended in 20 μl double distilled water. Quantitative PCR reactions were performed in presence of SYBR Green on ABI Prism 7000 gene expression system according to the manufacturers' instructions (Applied Biosystems, France) using 5-time dilution of each DNA. Bacteria were quantified using specific primers designed to amplified a 16S rDNA 150-bp-length fragment of *Blochmannia *(16SFor 5'-AGAATTCCAGGTGTAGCGGTG-3' and 16SRev 5'-TACGGCATGGACTACCAGGG-3'). Ant DNA were quantified using specific primers designed to amplify a 18S rDNA 150-bp-length fragment (18SFor 5'-TTAGAGTGCTTAAAGCAGGC-3' and 18SRev 5'-ACCTCTAACGTCGCAATACG-3'). These primers had been efficiently used in another study with *Blochmannia floridanus *[14]. The 18S rRNA ant gene copy number was used so as to normalize each dissected sample with the same quantity of ant DNA material. This gene was first specifically cloned and sequenced. Then real-time PCR specific primers (18SFor 5'-TTAGAGTGCTTAAAGCAGGC-3' and 18SRev 5'-ACCTCTAACGTCGCAATACG-3') were design based on the sequence and used to generate by classic PCR a 18S rDNA specific amplicon used to establish a standard curve expressing the Cycle Threshold (Ct) versus the logarithm of the copy number of 18S rDNA purified PCR products. These specific primers were also used to amplify 18S rDNA using DNA extracted from dissected samples. The exact copy number of 18S rDNA was established based on the experimentally obtained Ct value and the standard curve. This value was used to correct the calculated copy number of bacterial 16S rDNA.

#### Fluorescent In Situ hybridisation (FISH)

Bacteriocyte were visualized by FISH with oligonucleotide probes as previously described in the method topic "Symbiont identification".

## Authors' contributions

DJS and AL planned and coordinated the study. AB and DJS conducted the quantification of bacteria by q-PCR and FISH. DJS and DD identified the *Blochmannia*. All authors wrote the article.
